# Can microalgae grow on dissolved black carbon generated from high-frequency wildfires?

**DOI:** 10.3389/fmicb.2026.1777551

**Published:** 2026-02-20

**Authors:** Shah Faisal, Ahmad Mustafa, Mahdy Elsayed, Shangze Zhang, Xuyang Qiao, Irfan Saif, Javed Muhammad, Ting Li, Jialing Tang, Cassamo Ussemane Mussagy, Ayub Jadoon, Mian Gul Hilal, Ali Bahadur, Ashutosh Tiwari, Abdelfatah Abomohra

**Affiliations:** 1 Department of Environmental Engineering, School of Architecture and Civil Engineering, Chengdu University, Chengdu, China; 2 Faculty of Engineering, October University for Modern Sciences and Arts (MSA), Giza, Egypt; 3 Department of Agricultural Engineering, Faculty of Agriculture, Cairo University, Giza, Egypt; 4 MOE, Key Laboratory of Cell Activities and Stress Adaptations, School of Life Sciences, Lanzhou University, Lanzhou, Gansu, China; 5 Department of Microbiology, The University of Haripur, Haripur, Khyber Pakhtunkhwa, Pakistan; 6 Escuela de Agronomía, Facultad de Ciencias Agronómicas y de los Alimentos, Pontificia Universidad Católica de Valparaíso, Valparaíso, Chile; 7 Department of Microbiology, Abbotabad University of Science and Technology, Abbotabad, Pakistan; 8 Institute of Grassland Research, Chinese Academy of Agricultural Sciences, Key Laboratory of Biohazard Monitoring and Green Prevention and Control in Artificial Grassland, Ministry of Agriculture and Rural Affairs, Hohhot, China; 9 Cryosphere and Eco-Environment Research Station of Shule River Headwaters, Key Laboratory of Cryospheric Science and Frozen Soil Engineering, Northwest Institute of Eco-Environment and Resources, Chinese Academy of Sciences, Lanzhou, China; 10 University of Chinese Academy of Sciences, Beijing, China; 11 Institute of Advanced Materials, IAAM, Ulrika, Sweden; 12 Institute for Advanced Study, Chengdu University, Chengdu, China; 13 Aquaculture Research, AWI – Helmholtz Centre for Polar and Marine Research, Bremerhaven, Germany; 14 Aquatic Ecophysiology and Phycology, Institute of Plant Science and Microbiology, University of Hamburg, Hamburg, Germany

**Keywords:** aquatic system, black carbon, dissolved black carbon, light absorption, sustainability

## Abstract

Climate and land-use changes have significantly increased the severity and frequency of global wildfires, raising concerns about their effects on the terrestrial environment, aquatic systems, and humans. During wildfires, numerous substances such as organic matter black carbon (BC), anions, cations, and nutrients are released and mobilized. Black carbon (BC) is a pyrogenic residue generated through the incomplete burning of organics (OCs) during wildfires. The introduction of BC to aquatic systems through rainfall events forms a dissolved fraction known as dissolved black carbon (DBC), which strongly absorbs sunlight and increases both surface and internal water temperatures. Currently, microalgae are popular candidates for carbon fixation, biofuel production, and other value-added products. This review suggests the potential application of DBC in aquatic environments to enhance microalgal growth through sunlight absorption and interaction with other pollutants. However, the addition of DBC for microalgal growth may face challenges; therefore, the employment of novel strategies should be promoted to direct future research toward ensuring cleaner, more economical, and environmentally friendly DBC consumption for enhanced microalgal biomass production.

## Graphical Abstract

**Figure:**
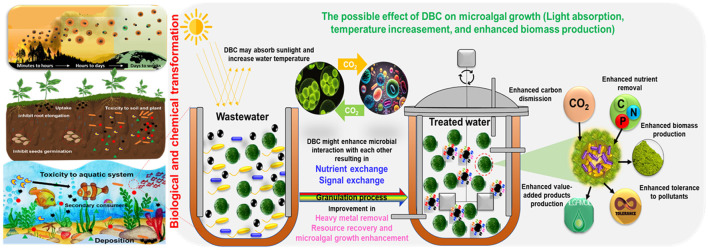

## Highlights

- Black carbon (BC) organic residues are formed by fossil fuel and biomass burning.
- Dissolved black carbon (DBC) affects the soil and aquatic system.
- DBC increases water temperature by absorbing sunlight, facilitating microalgal growth.
- Aquatic pollutants interact with DBC to mitigate its toxicity.
- The combination of microalgae and DBC could enhance green energy production.

## 1 Introduction

To modern society, “energy” is vital as it propels urbanization, economic expansion, and technological innovation (Roshan et al., 2023; Ercal and Shafique, 2026). To combat global warming, depletion of finite fossil resources, and fuel security, interest in renewable energy production is considerably increasing for sustainable development (Tiwari, 2024; Naqvi et al., 2025). Climate change mitigation and energy security serve as key drivers for the development of advanced sustainable technologies aimed at assessing diverse sustainability factors to attain efficient future energy systems (Tiwari et al., 2024; Naqvi et al., 2025). Biofuel production signifies an essential shift toward green energy sources, offering a sustainable alternative to traditional fossil fuels. By utilizing diverse biomass sources, including animal, algal, and plant materials, this approach leverages the natural breakdown capabilities of microbes to produce various types of biofuels, such as biohydrogen, biodiesel, biogas, and bioethanol (Thirumalaivasan et al., 2024; Han S.-F. et al., 2016). These biofuels play a significant role in promoting energy diversity and safeguarding the environment. First-generation biofuels, such as bioethanol and biodiesel, are produced by converting fats from animal and vegetable oils and fermenting sugars and starches derived from food crops. These fuels are recognized as alternatives to diesel and gasoline, contributing to the green energy landscape (Nisar et al., 2024). Second-generation biofuels, such as biogas, are produced from non-food biomass sources like lignocellulosic materials and agricultural waste. This approach helps address waste disposal issues and enhances the value of agricultural by-products. Third-generation biofuels expand the range further by utilizing efficient energy converters, such as microalgae (Thirumalaivasan et al., 2024). Microalgae are excellent sources of biofuel due to their high levels of carbohydrates, lipids, and proteins, which are essential for biofuel production. The choice of microalgae species depends on their specific compounds: those with high lipid content are best for producing biodiesel, while species rich in carbohydrates are more suitable for bioethanol and biogas (Nagappan et al., 2019).

Global warming is a critical environmental crisis that requires urgent action. As Earth's climate rapidly shifts, it is essential to assess the effects of global warming and implement robust measures to mitigate its potentially serious consequences. Scientific consensus is unanimous: Human actions, especially deforestation and the extensive use of toxic chemicals and their derivatives, are primary drivers (Son et al., 2025). Global warming increases the risk of wildfires and intensifies greenhouse gas and pollutant emissions (Cunningham et al., 2024). Recent wildfire outbreaks worldwide have raised concerns that climate change is increasing fire frequency, endangering human livelihoods and biodiversity, and exacerbating climate change (Jones et al., 2022). Fire is a crucial factor in shaping ecosystem processes (McLauchlan et al., 2020); additionally, it transfers carbon from the biosphere into the atmosphere through the burning of plant biomass, surface litter, and even soil organic matter (Van Der Werf et al., 2017; Zheng et al., 2023).

**Figure 1:**
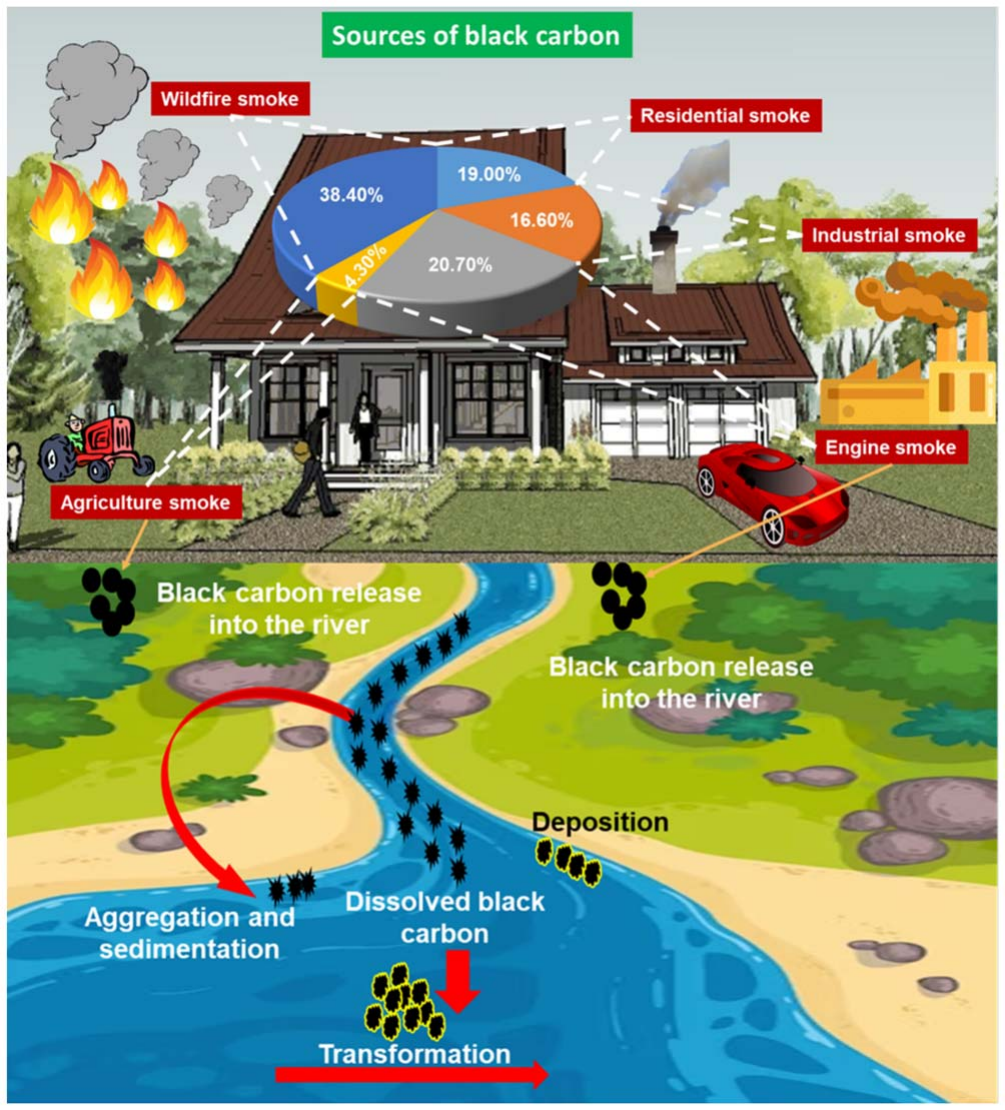
Black carbon generation sources and introduction routes to the ecosystem.

During wildfires, pyrogenic carbon (PyC), which includes particles of various sizes and compounds from pyrogenic sources such as charred biomass, black carbon (BC), and soot, is generated concurrently through the partial combustion of biomass resources (Preston and Schmidt, 2006; Bird et al., 2015). Worldwide estimates of particulate organic carbon and BC release from combustion indicate that about 88% of the entire carbonaceous aerosol mass comes from biomass burning (Bond et al., 2004; Sengupta et al., 2018). Among the major sources of emissions, biomass burning is a notably prominent contributor, significantly affecting regional air quality (Zhou et al., 2017; Chen et al., 2017; Vicente and Alves, 2018; Liu et al., 2018; Nahak et al., 2022). Structurally, it is composed of quasi-spherical primary particles that range in diameter from 10 to 100 nm and are likely to aggregate and form more complex agglomerates. BC includes a range of carbon-based materials produced through the burning of fossil fuels and the partial combustion of biomass, particularly in wildfires (Fan et al., 2025). BC sequesters carbon in both marine and terrestrial habitats, making it ecologically persistent for hundreds to thousands of years. To date, it is not well understood how it is produced, moves, is stored, and contributes to the global carbon cycle (Coppola et al., 2022). The release of BC to soil, sediments, the atmosphere, and the marine environment can occur in the forms of particulate black carbon and dissolved black carbon (DBC) (Fan et al., 2025; Figure 1).

The remobilized portion of BC is known as DBC, and its main composition is based on functional groups of hydrophilic oxygen, including phenolic and carboxylic groups, along with aromatic fused rings (Fu et al., 2018; Wagner et al., 2019; Qi et al., 2020; Coppola et al., 2022). The dominant portion of the inert carbon pool in the marine environment consists of DBC derived from BC, which plays a vital role in the carbon cycle within the atmosphere, in the ocean, and at the interface of the oceans and atmosphere (Hameed et al., 2023). DBC derived from the terrestrial ecosystem highlights an important relationship between terrestrial and aquatic systems (Hameed et al., 2023). The introduction of DBC to the marine environment serves as an important intermediary in the global carbon cycle. Previous studies show that DBC stimulates microbial metabolism more efficiently than the DBC flux from rivers and exports a significant portion of reduced carbon to the marine environment from the surface, strengthening the biogeochemical cycling of coastal boundaries (Yamashita et al., 2021; Mukherjee and Kumar, 2021). Worldwide, the DBC flux in riverine systems corresponds to 10% of the total oceanic dissolved organic carbon (DOC), varying in marine environments from 4% to 22% DBC and from 4% to 7% in coastal marine areas (Chen et al., 2022). As shown in Table 1, diverse water sources exhibit changing DBC levels.

**Table 1:** Concentration of dissolved black carbon in different regions.

**Site**	**DBC Concentration (μM)**	**References**
East China Sea	2.6 ± 0.8	Park et al., 2025
Yellow Sea	2.2 ± 0.1	Park et al., 2025
East Sea	1.3 ± 0.1	Park et al., 2025
Pacific Ocean	0.5 ± 0.1	Yamashita et al., 2022
Bering Sea	1.5 ± 1.0	Fang et al., 2021
Canada Basin	1.4 ± 0.3	Fang et al., 2021
Chukchi Sea	1.3 ± 0.2	Fang et al., 2021
Bohai Sea	6.7 ± 2.5	Fang et al., 2020
Mariana Trench	3.6 ± 2.4	Qi et al., 2020
Atlantic Ocean	0.8 ± 0.0	Wagner et al., 2019
Prydz Bay shelf	0.8 ± 0.3	Fang et al., 2018
South China Sea	1.0 ± 0.2	Fang Z. et al., 2017
Beaufort Sea	2.2 ± 0.6	Coppola and Druffel, 2016

Nevertheless, the DBC circulation across various marine systems and its implications for safe drinking water have not been extensively studied (Chen et al., 2022). The antagonistic effects of DBC on aquatic ecosystems, such as water pollution and human risk, have been widely discussed; however, the effect of DBC on microalgae remains a concern and is a future perspective. Therefore, this review article aims to highlight the effect of DBC on aquatic ecosystems, plants, and human health. Additionally, the role of DBC and SWBC in the growth of microalgae is suggested. The interaction between DBC and other contaminants in relation to their effects on microalgae growth is evaluated.

## 2 BC detection techniques

BC identification and quantification are essential for assessing its biological and ecological impacts. However, standardized methods for analyzing BC are lacking because of its ambiguous definition. The investigation of BC encompasses various disciplines, including toxicology, atmospheric science, marine science, and soil science, and descriptions of BC vary across these fields. For instance, BC is commonly called soot in combustion science, resulting from the pyrolysis of hydrocarbons or incomplete combustion, and it likely produces bright light when exposed to laser heating at elevated temperatures (Michelsen et al., 2020; Martin et al., 2022). In atmospheric science, these are referred to as carbonaceous particles with strong visible light absorption, including soot, oil char, and coal dust (Petzold et al., 2013; Karanasiou et al., 2015). In toxicological sciences, BC refers to carbonaceous particles, as in atmospheric sciences; however, toxicologists are more concerned with the slightly smaller soot particles because of their widespread presence and significant implications for health risk assessments (Janssen et al., 2011; Kuntic et al., 2022). In the domains of oceanic and soil sciences, BC refers to carbonaceous materials that are resistant to oxidation and demonstrate persistence in soils and sediments. In soil, typically more than 60% of the organic matter from partially combusted products is assigned to BC (Masiello, 2004). The differences in the description of BC are based on varying focuses across disciplines, resulting in distinct requirements for analytical methodologies related to different media. The approaches to the analysis of BC also vary based on the field and samples. The determination of BC methods may be classified into four categories based on the instruments and principles employed: chemical analysis, mass spectrometry, optical techniques, and microscopic techniques (Table 2).

**Table 2:** BC analysis methods overview across diverse media including the sample representation.

**Category**	**Technique**	**Sample**	**Sample preparation**	**Volume**	**Media**	**Advantages**	**Limitations**	**References**
Microscopy	Scanning electron microscopy (SEM) and Transmission electron microscopy (TEM)	Aerosol Soil Lung	Ultrasonic extraction Ultrasonic extraction Separation of coarse components Tissue fixation and sectioning	Few	All media	Simple operation and Equipment High qualitative Ability Few sample volumes	Not able to quantify Susceptible to interference in a complex matrix Only observe a limited number of particles	(Brodowski et al., 2005; Chen et al., 2005; Liati et al., 2014; Pabst and Hofer, 1998; Wensing et al., 2011)
	PPPM	Urine	Not specified	Few	Urine water	Imaging analysis Few sample volumes	Expensive instruments Quantitative inaccuracy Not useable for organic-rich substrates	(Steuwe et al., 2018)
	Femtosecond pulsed laser microscopy (FPLM) FPLM	Urine Placenta Buffer medium	Not specified Tissue fixation and sectioning Ultrasonication to break aggregates Vortexed properly suspend the particles	200 μL Few 250 μL	Urine water blood tissue	Low detection limit Imaging analysis Few sample volumes	Expensive instruments Not useable for organic-rich substrates Interference from Particle aggregation	(Aslam and Roeffaers, 2021; Bové et al., 2019; Saenen et al., 2017)
Optical methods	AE51	Aerosol	*In situ* measurement	Online	Aerosol	Simple operation and equipment; *In situ* and real-time monitoring	Interference caused by light-absorbing substances	(Kar et al., 2012)
	OT21 OT21	Aerosol Snow	*In situ* measurement Melting and filtering of snow samples	Not specified 500 mL	Aerosol Snow Ice Water	Simple operation and equipment *In situ* analysis Low detection limit	Interference caused by light-absorbing substances	(Ahmed et al., 2009; Cereceda-Balic et al., 2019)
	Photoacoustic sensor	Aerosol	*In situ* measurement	Online	Aerosol	Simple operation and equipment *In situ* and real-time monitoring	Low analytical sensitivity	(Beck et al., 2003)
	TOT TOR TOT	Aerosol Aerosol Snow	*In situ* measurement *In situ* measurement Melting and filtering of snow samples	Not specified Not specified 100 mL	Aerosol Snow Ice Water	*In situ* analysis Multimedia analysis Simultaneous analysis of multiple black carbon species	Potential sample loss during sample transfer Filter membrane effects	(Hou et al., 2011; Lim et al., 2014; Liu et al., 2019)
	Single particle soot photometer (SP2)	Snow Aerosol	Sample melting and aerosolization *In situ* measurement	50 mL Online	Snow Ice Aerosol	*In situ* and real-time monitoring Low detection limit	Limited detection range (70–500 nm) Inorganic salts interference effect	(Zanatta et al., 2021; Zhang et al., 2021)
	Raman	Aerosol	Ultrasonication	Not specified	All media	Simple operation and equipment	Low analytical sensitivity Matrix interference	(Wang et al., 2021)
Mass spectrometry	Laser desorption ionization mass spectrometry (LDI-MS) LDI-MS	Mice organs Aerosol	Tissue homogenization *In situ* measurement	1 μL 0.28 cm^2^ membranes	Tissue Aerosol Water	*In situ* analysis Imaging analysis Few sample volumes Multimedia analysis Low detection limit	Expensive instruments	(Lin et al., 2021; Min et al., 2022)
	Secondary ion mass spectrometer (SIMS)	Aerosol	Not detected	Not detected	Aerosol	Imaging study *In situ* study High spatial Resolution	Expensive instruments Not suitable for large volume samples or bulk Phase determination	(Cheng et al., 2014)
	Accelerator mass spectrometry (AMS)	Aerosols	*In situ* measurement	Online	Aerosols	*In situ* and real-time monitoring Low detection limit Low detection limit	Not suitable for samples that are difficult to nebulize	(Kirchner et al., 2003; Lee et al., 2015)
Chemical analysis methods	Wet chemo oxidation	Soil	Acidification with 10 % HF for 12 h	250–400 mg	Soil Sediment	Simple equipment Multimedia analysis	High detection limits Coking effect; Impurity interference	(Knicker et al., 2007)
	CTO-375	Soil	Acidification with 12 M HCl for 4 h	5–25 mg	Soil Sediment	(1) Simple equipment (2) Multi-media analysis	High detection limits Complex pretreatment procedures Not applicable for low- condensed black carbon Coking effect	(Agarwal and Bucheli, 2011; Eckdahl et al., 2022)
	BPCAs	Soil	Acidification with 4 M trifluoroacetic acid Residue was collected by filtration and oxidized with 65 % HNO_3_ for 8 h at 170 ?C	0.5 g	Water Soil Sediment	Multi-media analysis Low detection limit The capability of analysis of dissolved black carbon	Complex pre-treatment procedures Not applicable for low-condensed black carbon	(Llorente et al., 2018)

In early 2007, 17 laboratories across different disciplines collaborated to investigate 12 distinct sources of BC utilizing seven kinds of analytical techniques (Hammes et al., 2007). This collaboration revealed a significant discrepancy in the analysis across all disciplines. For instance, atmospheric scientists prioritized online monitoring techniques, employing optical and mass spectrometry techniques such as SP2, soot particles aerosol mass spectrometry, and light absorption approaches (Petzold et al., 2013). In contrast, marine and soil scientists often use offline bulk sampling methods for BC analysis, while chemical analysis techniques, including wet chemical oxidation and chemo thermal techniques, are major choices for soils and sediments (Masiello, 2004). However, quantifying DBC in the marine environment is quite challenging using the above-mentioned chemical analysis procedures, which are typically determined through benzene polycarboxylic acids methods (Chen et al., 2022).

Recent findings indicate that exposure to BC correlates with numerous cardiovascular and respiratory illnesses, highlighting an urgent need for analytical techniques to quantify BC in biological samples. These techniques include LDI-MS, photothermal pump-probe microscopy, FPLM, and Raman spectroscopy (Dittmar and Paeng, 2009; Bové et al., 2016; Steuwe et al., 2018; Lin et al., 2021). The variability in methods and standards for BC analysis complicates the comparison of analytical results across different disciplines. Furthermore, certain recently developed analytical methods, such as LDI-MS, have the capability to be utilized across various media, though their adoption has been limited.

## 3 Effect of BC and DBC on the environment, humans, and plants

**Figure 2:**
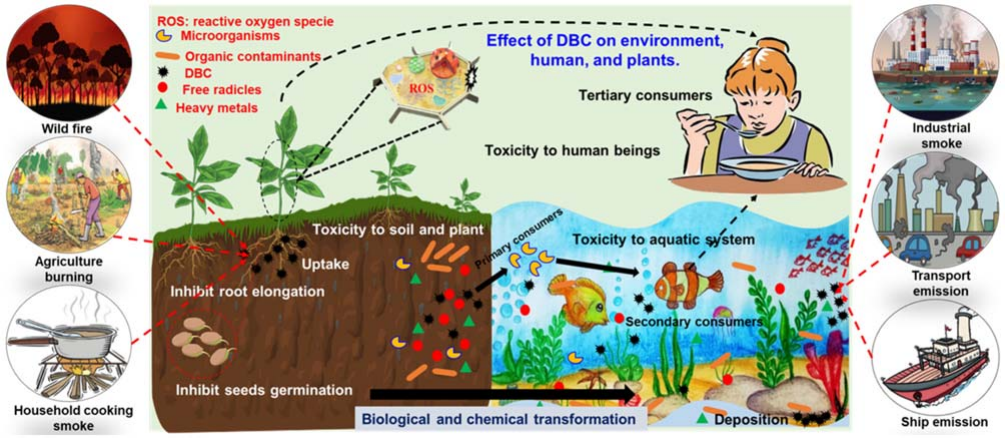
The potential detrimental impact of DBC on the ecosystem (soil, flora, aquatic species, and humans).

Currently, the majority of research emphasizes the advantages of BC usage for crop cultivation (Hussain et al., 2016; Mansoor et al., 2021) and in soils (Guo et al., 2021), with minimal investigation into the associated risks of BC (Mumme et al., 2018; Godlewska et al., 2021). Researchers have identified that DBC produced from BC is vulnerable to the effects of naturally aged soil rich in nutrients, which might modify its properties (Yang et al., 2021; Xu et al., 2021). Figure 2 illustrates the detrimental influences of BC on soil, organisms, and plants. During the pyrolysis process, mobile organic compounds (OCs) from BC cannot be separated, which may lead to the generation of large quantities of potentially harmful organics, such as short molecular weight phenols, volatile organic compounds, alcohols, and ketones (Cordella et al., 2012). In the meantime, the release of polycyclic aromatic hydrocarbons occurs with DBC after the application of BC (Sfetsas et al., 2011). Similarly, the low molecular weight of DBC, which contains a hydrophilic fraction, could easily bind with organic soil components to produce soluble complex molecules and occupy the active binding sites within the soil biosystem (Liu et al., 2019). This would consequently result in a reduction of the binding sites in the soil responsible for the adsorption of harmful pollutants, such as Hg^2+^ and Cd^2+^ (Liu et al., 2019), consequently enhancing the mobility of these pollutants (He et al., 2019; Wang et al., 2020). Furthermore, DBC enhances the release of harmful elements by influencing the solubility capacity of sandy soil minerals, such as Mg^2+^ and Fe^2+^, which primarily act as binding sites for oxyanion pollutants through ligand-exchange complexation, resulting in an enhancement of metallic elements' movement into the soil (Wang et al., 2017).

DBC functions as a facilitator in marine and terrestrial environments, enhancing the movement of pollutants. The interaction of DBC may block pores and reduce the attachment sites for OCs such as atrazine (Kasozi et al., 2010) and heavy metals like Cu^2+^ (Ahmad et al., 2014), thereby accelerating the movement and transportation of pollutants in soil as well as in groundwater, consequently increasing contamination risks to both aquatic organisms and soil. The emission of harmful organics can damage plant cells and reduce seed development and germination (Mumme et al., 2018). The osmotic pressure (salinity from available K) and pH adversely affect plant development in mineral-rich BC (Buss et al., 2016). BC treatment has been shown to inhibit microbial growth (Eo et al., 2018), reduce dominance and production (Liu et al., 2019), adversely alter structural integrity, and induce cytotoxicity and neurotoxicity (Flesch et al., 2019). The development and even the existence of flora and fauna might be negatively affected by aged BC (Anyanwu et al., 2018), as well as the soil microbial flora (Liu et al., 2015). Furthermore, the generation of harmful DBC from pinewood, which potentially contains lignin mono-, di-, and tri-phenolic derivatives, contrasts with DBC derived from BC that originated from cellulose, which has an acidic nature and bio-oil components. Moreover, DBC from rice straw BC has the ability to generate free radicals, which further produce OH radicals, resulting in the formation of reactive oxygen species (ROS) that may inhibit seed germination or adversely influence the development of rhizomes in crops (Feng et al., 2021), affecting nematodes and microalgae (Lieke et al., 2018). In this context, the DBC concentration of 280.0 mg DBC/kg BC was determined to be potentially toxic for blue–green algae *Synechococcus* sp. of freshwater (Smith et al., 2016).

(Huang et al. 2019) reported that exposure to 300 mg/L DBC induces oxidative stress in indigenous microorganisms, resulting in irreversible damage to organelles. Recent investigations have observed the risk of morbidity and mortality associated with prolonged exposure to BC (Hvidtfeldt et al., 2019; Ljungman et al., 2019). Nonetheless, the outcomes of these studies were predominantly consistent. For example, 2.4 million Canadians aged 25 or older with prolonged exposure to BC exhibited a significant association with mortality (Crouse et al., 2016). (Ostro et al. 2015) identified a significant correlation between BC and mortality rates from ischemic heart disease, but not with overall mortality rates. BC particles migrate to the human brain and accumulate in key regions, such as the frontal and temporal lobes, which are involved in emotional regulation (Vanbrabant et al., 2024). Previous studies have established that BC can adsorb carcinogenic pollutants such as polycyclic aromatic hydrocarbons (PAHs). It can also penetrate deeply into the human respiratory system, increasing the risk of childhood asthma, lung function decline, and stroke (Boniardi et al., 2021; Cao et al., 2012; De Prins et al., 2014; Wei et al., 2015). Bové et al. (2019) found that BC particles gather on the fetal side of the placenta, suggesting that ambient particulates might be carried toward the fetus and represent a possible mechanism explaining the damaging health effects of pollution from early life onwards. The structural features of DBC and the composition of contaminants determine whether DBC derived from BC has a positive or negative impact on soil. Consequently, it is essential to evaluate the correlation between external factors and the characteristics and toxicity levels of BC-DBC.

## 4 Accumulation of BC on surface water and DBC

BC is characterized as a material composed of condensed secondary compounds, exhibiting chemical stability and limited biodegradability (Landry and Matthews, 2017; Abney and Berhe, 2018). Suman et al. (1997) reveal that decreasing particle size to less than 2 mm enhances the airborne retention of BC granules, facilitating long-distance dissemination. Immediately following fires, generated BC particles over 1 μm in size may not become airborne or may rapidly settle onto the adjacent surface (Clark and Patterson, 1997; Masiello, 2004). Precipitation and runoff mechanisms may ultimately transport such BC particles from the soil surface into rivers and seas unless deposition occurs more than 1 km from significant water bodies (Clark and Patterson, 1997). Larger BC particles and combusted materials may not be transported and may instead persist in the soil at or near the location of their production and deposition. Recent investigations indicate that DBC may constitute a substantial fraction of BC mobilization and its transportation by rivers to the ocean (Ziolkowski and Druffel, 2010; Jaffé et al., 2013; Coppola et al., 2018). Jaffé et al. (2013) reported that each year, global rivers receive over 27 million tons of DBC, accounting for 10% of the global flux of dissolved organic carbon (DOC). Coppola et al. (2019) indicated that the quantity of DBC conveyed by rivers is similar to the global transport of PBC, estimated at approximately 17–37 million tons annually. The annual delivery of DBC and PBC by rivers may account for 8%−60% of total yearly BC output, establishing it as a significant contributor to BC input in the ocean. Dittmar et al. (2012) investigated the seasonal variability of riverine DBC from 1997 to 2008 in tropical Atlantic Forest watersheds that had experienced substantial burning from 1850 to 1973. They noted that even after a widespread forest fire that ended more than 20 years ago, DBC continues to be transported from the watershed each year during the rainy seasons. Marques et al. (2017) demonstrated that hydrology plays a significant role in the DBC breakdown and mobility, as peak DBC levels were observed during the rainy season and minimal concentrations during the dry season of the same river.

Due to its dense cyclic aromatics, DBC is a hydrophobic organic compound (Qu et al., 2016). Consequently, particulate DBC (PDBC) can be found on suspended particles or granular organic substances. Numerous methodologies, such as atom/fragment function and FT-ICR-MS, are currently employed in research to infer the octanol–water partitioning coefficient of DBC. Wagner et al. (2017) posited that DBC, characterized by highly concentrated structures, possesses a high octanol–water partition coefficient, indicating that a significant fraction of DBC in the watershed remains unresolved. Geng et al. (2021) detected DBC and other dissolved organic compounds in coastal air aerosols in Southeast Asia. Liu et al. (2019) noted that DBC can adhere to fine particles through vigorous substitutions of functional groups at the soil interface and co-sorption with the soil's intrinsic carbon molecules. Coppola et al. (2014) discovered that DBC immobilized on floating particles and embedded in deep sea sediments constitutes a substantial DBC sink within marine ecosystems.

DBC in the marine environment is commonly defined as the fraction of black carbon (BC) capable of passing through standardized filtration media (Qu et al., 2016; Li et al., 2019). The presence of DBC in surface waters is largely associated with atmospheric deposition and terrestrial runoff processes, during which BC particles undergo chemical transformation, dissolution, and redistribution among environmental compartments (Burkhard et al., 2008; Zigah et al., 2012). Increasing fossil fuel consumption, along with the documented rise in wildfire frequency and severity driven by climate warming (Abatzoglou and Kolden, 2011; Iglesias et al., 2022), is expected to significantly elevate DBC levels in surface waters, particularly in regions prone to fire. Prolonged fire seasons and enhanced aridity have already contributed to longer-lasting and more intense wildfires in recent decades (Liu et al., 2010; Jolly et al., 2015). Wildfire events act as major sources of BC, releasing large quantities into the atmosphere where they can be transported over considerable distances. Observational studies have consistently linked higher atmospheric BC levels to wildfire activity occurring far from the point of emission (Iglesias et al., 2022; Ahlberg et al., 2023). Once deposited onto aquatic surfaces or surrounding catchments, these BC particles may dissolve, thereby contributing to the pool of DBC in surface waters (Burkhard et al., 2008; Wagner et al., 2018). As a result, the worldwide increase in wildfire activity has the potential to alter surface water DBC dynamics on a broad spatial scale.

The pronounced light-absorbing and heat-trapping characteristics of atmospheric BC prompt consideration of whether similar effects may occur once BC becomes dissolved in aquatic environments. Comparable phenomena have been observed in snow-covered regions, where BC deposition decreases surface albedo and accelerates snowmelt through enhanced absorption of solar radiation (Flanner et al., 2007; Kaspari et al., 2015). A similar response might be expected in surface waters containing elevated DBC concentrations. Because BC is produced under a wide range of combustion conditions and from diverse organic precursors (Baldock and Smernik, 2002; González-Pérez et al., 2004), DBC encompasses a heterogeneous mixture of molecular structures, spanning from low-aromatic, carbohydrate-like compounds to highly condensed polyaromatic forms (Wagner et al., 2018).

**Figure 3:**
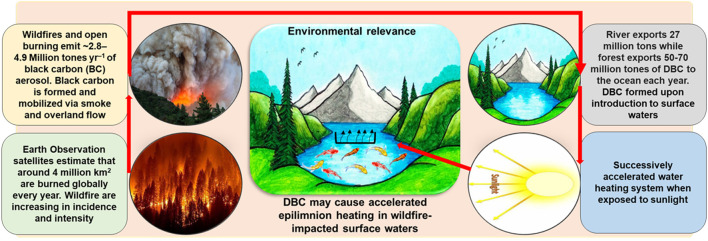
Black carbon (BC) and dissolved black carbon (DBC) as a possible driver of surface water heating dynamics in wildfire-affected regions.

The molecular composition of DBC is not static but evolves through exposure to photochemical degradation (Fu et al., 2016; Li et al., 2019), microbial processing (Baldock and Smernik, 2002; Hamer et al., 2004), and physicochemical interactions such as aggregation or precipitation that depend on local aquatic conditions. These transformation pathways collectively influence the optical properties of DBC and may modify its contribution to light absorption and thermal processes within the water column. Although DBC typically represents a relatively small proportion of total organic carbon (TOC) or DOC in several aquatic systems (Wagner et al., 2018), its strong absorptive capacity suggests that it may exert a significant influence on water heating (Figure 3). Dissolved organic matter is recognized to absorb radiation across ultraviolet and infrared wavelengths, with maximum optical activity often occurring near 254 nm due to aromatic functional groups (Weishaar et al., 2003). While DBC is generally less aromatic than its original biomass-derived BC precursor (Qu et al., 2016), it remains more aromatic than most other dissolved organic constituents in aquatic environments (Wagner et al., 2017). This relative enrichment in aromatic structures indicates that DBC may play a notable role in absorption-driven thermal dynamics in surface waters.

## 5 BC and DBC in the marine environment

Wildfires are the main source of black carbon (BC) to the environment, and the vast majority of BC is generated from deposits on the landscape, while other sources include the combustion of fossil fuels and the burning of biomass (Santín et al., 2016; Reisser et al., 2016). The BC generated from the burning of fossil fuels is the predominant source in the atmosphere, known as aerosol BC; however, its annual concentration is lower than that deposited in the soil (Bird et al., 2015). The initial presence of DBC in natural marine environments was identified as condensed aromatic compounds in volcanic ash, soil humic acid, pre-waters, and in the waters of Rio Negro through ultrahigh-resolution mass spectrometry (Kramer et al., 2004; Hockaday et al., 2006; Kim et al., 2004). Soil extracts contained condensed aromatic compounds ranging from 110 to 145 ppm, measured through nuclear magnetic resonance (NMR) analysis (Moller et al., 2000), which were also reported in dissolved organic matter in the ocean (Dittmar and Koch, 2006). The transport of DBC from soil to water and the ocean shows terrestrial BC sequestration and contributes to marine DBC (Dittmar and Paeng, 2009). Research findings showed that BC influenced the marine niche and disrupted the global carbon cycle (Mari et al., 2014; Shiu et al., 2014). BC demonstrated a strong affinity for many persistent environmental contaminants due to its aromatic structure and acts as a vector, carrying them to the surface and marine water columns and sediments (Bucheli and Gustafsson, 2000; Lohmann et al., 2009; Lian and Xing, 2017; Alam et al., 2018; Luo et al., 2019; Xu et al., 2021).

Recently, research on marine DBC has increased due to high concentrations (0.1%−7%) (Wagner et al., 2018). The old deposited DBC can be determined through ^Δ^14C isotopic analysis (Ziolkowski and Druffel, 2010; Coppola and Druffel, 2016). It has been suggested that DBC significantly contributes to persistent DBC and thus reduces the conversion of DOC in the marine environment (Coppola and Druffel, 2016). Although riverine and atmospheric DBC are recognized as major sources of marine DBC (Bao et al., 2017; Wagner et al., 2018), the δ13C signature of ocean DBC is distinct from that of streams, indicating unaccounted sources of marine DBC (Wagner et al., 2019). Different toxic and recalcitrant compounds, such as furans, dioxins, volatile organics, and polycyclic aromatic hydrocarbons (PAHs), can be produced during BC carbonization (Keiluweit et al., 2012; Kołtowski and Oleszczuk, 2015; Lyu et al., 2016; Lian and Xing, 2017). BC and DBC are toxic to many organisms, including plants, animals, aquatic algae, *Caenorhabditis elegans, Daphnia magna, Pseudomonas aeruginosa*, and *Triticum* spp. (Kołtowski and Oleszczuk, 2015; Smith et al., 2016; Yang et al., 2017; Sigmund et al., 2017; Lieke et al., 2018; Li et al., 2019; Hill et al., 2019; Godlewska et al., 2021). DBC exhibits a strong affinity for various compounds like chlorinated benzenes, PAHs, and other organic compounds, as well as heavy metals such as Cu, Cr, and Cd, owing to its aromatic structure and oxygen-containing functional groups (Qian et al., 2016; Fu et al., 2018; Huang et al., 2019; Lian et al., 2019; Xu et al., 2020). This suggests that DBC may act as a Trojan vector for many contaminants (Lian and Xing, 2017; Xu et al., 2020, 2021). Thus, it is crucial to understand the fate of DBC in water, as this would help in toxicological analysis as well as the behavior of attached pollutants.

### 5.1 Biocarbon-based materials as the mainstay for microalgae cultivation

During the past few years, biocarbon-based technologies have emerged as a new, economical, and environmentally neutral approach to treating industrial effluents, as well as showing promise in desalinating seawater. Their renewable nature provides both economic and environmental benefits, making them a potential solution for water pollutants. Various studies have shown that activated carbon, biochar, and biocarbon synthesized in laboratories can be effectively used as adsorbents for various organic pollutants (Rosales et al., 2017; Yuan et al., 2022). The physical and chemical properties of these biocarbon-based materials, such as large surface area, ubiquity in the environment, vast microporosity, high adsorption capacity, and strong structural integrity, make them especially useful in wastewater treatment (Chausali et al., 2021). In this context, microalgae, including eukaryotic algae and cyanobacteria, represent a diverse, multifunctional assembly of microorganisms, characterized by their ability to capture CO_2_ via photosynthesis and, thus, produce an array of algal cellular components, energy, and oxygen. They have become a stable and environmentally friendly alternative to traditional, energy-consuming methods of microbiological treatment that are currently predominant (Faisal et al., 2022b; Abomohra and Ende, 2024).

The last two decades have seen significant industrial and academic effort in the process design methodology of energy conservation and waste reduction across a wide variety of manufacturing industries worldwide (Ebaid et al., 2019; Faisal et al., 2022a; Wang et al., 2022; Soni et al., 2024). Microalgae present promising opportunities in the overall range of potential applications of green biotechnology, such as chemical synthesis and the creation of various green energy vectors, which offer significant environmental benefits (Shao et al., 2019). As one of the oldest known photosynthetic systems on the planet, observable in both marine and terrestrial environments, these organisms possess a distinctive capacity to obtain and exploit CO_2_. The process of using CO_2_ and sunlight can result in much higher growth rates and 10–50 times greater carbon fixation rates than terrestrial plants (Xu et al., 2023). The use of wastewater to grow microalgae has been studied as a feasible approach to lowering production costs and commercializing microalgal biomass in recent years, with pilot facilities successfully established (Han S. et al., 2016; Elshobary et al., 2019). It is worth mentioning that the growth and biodiesel production rates of microalgae when cultivated in municipal wastewater are high (Han S. et al., 2016). Currently, the challenge of growing microalgae in full-strength biogas slurry is linked to high levels of nutrients like organic matter and ${\text{NH}}_{4}^{+}$N, which negatively impact microalgal growth (Gu et al., 2021). For example, anaerobic digestate or textile wastewater with high amounts of ${\text{NH}}_{4}^{+}$N can cause depolarization of the cell membrane in microorganisms, inhibiting intracellular anion transport and ultimately leading to disturbances in growth and metabolism (Ritchie, 2013). Free fatty acids in anaerobic digestate have cytotoxic effects on the cytoplasmic membranes of some microalgal species, disturbing membrane permeability, causing potassium leakage, and leading to cell lysis (Wu et al., 2006).

Behl et al. (2019) explored the concept of using biochar as an additional nutrient for growing *Chlorella pyrenoidosa* in an aqueous solution of Direct Red 31. They evaluated the resultant de-colorization of dye, algal biomass, and lipid content. The results indicate that biochar made from sawdust may be an economical bio-nutrient for cultivating algae and biodiesel, while also being effective in the treatment of textile effluents. Wang et al. (2023) investigated the growth of *Chlorella protothecoides* in the presence of tetracycline, with biochar added during the growing stage. The addition of biochar improved algal biomass growth by 13.26 percent compared to algal cells cultivated in the absence of the biochar. Although biochar showed partial elimination of tetracycline on its own, when combined with *C. protothecoides*, it resulted in complete removal of the antibiotic.

**Figure 4:**
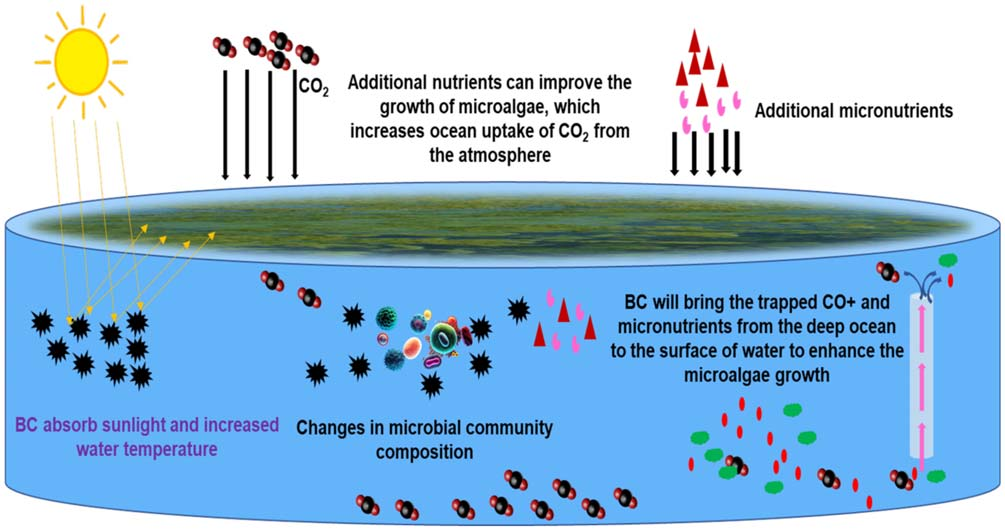
Biocarbon-based materials can bring trapped carbon dioxide and micronutrients from the deep ocean, which may facilitate the growth of microalgae (Proposed).

The study highlights the significance of the combination of biochar and microalgae to achieve efficient tetracycline removal and justifies their potential to protect the environment (Figure 4). Bio-based materials, as well as algae, have the ability to eliminate pollutants, and combining both represents a new approach that may enhance the effectiveness of these materials in the elimination of pollutants from water. However, practical application is not an easy task. For example, biocarbon is often found in a powder or granular state, whereas algae are typically suspended in water. This difference in structure may result in the loss of material during treatment and further complicate the reuse of the substance after depletion (Zheng et al., 2017). To address these challenges, a novel technique has been implemented to fix the algae and biocarbon simultaneously, thereby synergistically combining their natural capabilities to eliminate water contaminants.

### 5.2 DBC effect on microalgae

**Figure 5:**
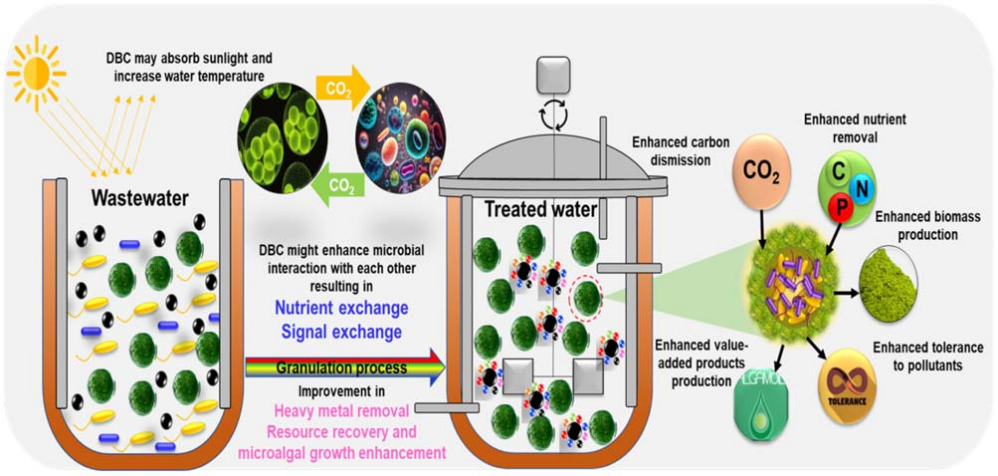
The possible effect of dissolved black carbon on microalgal growth (light absorption, temperature increase, and enhanced biomass production). Dissolved black carbon can serve as a habitat for pollutants, and microalgae can use both pollutants and DBC as growth enhancers for renewable energy production.

In marine environments, the main source of DBC entering surface waters is identified as the discharge from overland flow and atmospheric deposition (Zigah et al., 2012). In addition, the continued burning of fossil fuels and an expected increase in the frequency of wildfires—estimated at a 14% increase in global extreme fires by 2030, 30% by the end of 2050, and 50% by the end of the century associated with enhanced climate warming (Wuebbles et al., 2017; Iglesias et al., 2022)—would likely lead to higher concentrations of DBC in surface waters following fires on decadal timescales in fire-disturbed regions. The light-absorbing and heating properties of BC in the atmosphere prompt interest in whether DBC can impart similar absorptive and heating characteristics to the surface waters where it deposits. Similar to biochar, the influence of DBC on microalgal growth can also depend on multiple factors. Microalgae have specific environmental requirements, and alterations in water chemistry might affect their growth. Being highly aromatic, DBC may participate in several physicochemical processes with coexisting nutrients, influencing their availability to microalgae (Figure 5). Such adsorption can be either favorable or detrimental, depending on the nutritional needs of the particular strain of microalgae. DBC has potential applications as a source of carbon for microorganisms, such as microalgae. Some microalgae can utilize dissolved organic carbon for growth, and DBC can be a part of the aquatic carbon pool. DBC could affect the microbial community in water bodies, and relationships between microorganisms might be disrupted (even indirectly due to ECM), impacting microalgal growth. DBC may also affect the overall water chemistry (e.g., pH, conductivity). The complex interactions of DBC with other substances in the aquatic environment and the pollutant mixture are intricate and need to be fully evaluated to predict their potential environmental impacts.

The inhibitory impact of DBC on microalgal growth was assessed by comparing microalgal growth cultivated in BG-11 medium, DBC400, and DBC600 at concentrations of 61.9 wt%. Notably, DBC400 and DBC600 promoted microalgal growth, with the biomass of *R. robustum* increasing to 6.3 and 5.9 g/L after 96 h of exposure, respectively, compared with the findings of Huang et al. (2020), which differ from previous results. In another investigation, biochar was used to evaluate the growth of *Anabaena cylindrica* and *Klebsormidium flaccidum* cultures utilizing BG-11 on a solid support, compared with the growth of a control. During a 20-day incubation under a light-dark cycle of 16:8, the total nitrogen, carbon, and dry biomass contents were assessed in cultures with biochar and in the control without biochar solid support. The *A. cylindrica* culture showed an approximate 80% enhancement with biochar compared to the control culture without biochar. In addition, a 10% increase in nitrogen content was observed in the material collected from the *A. cylindrica* culture with biochar solid support (Kholssi et al., 2018).

## 6 DBC interaction with other pollutants

**Figure 6:**
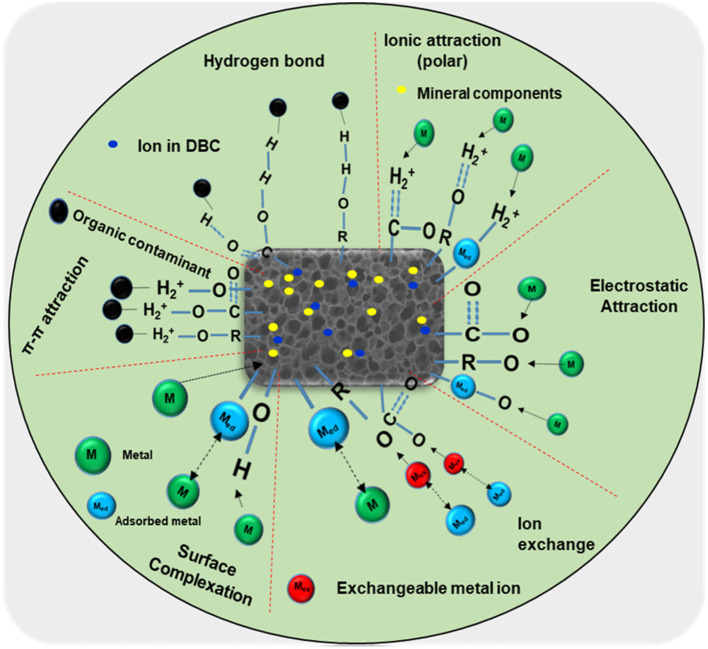
Mechanisms and interactions of DBC with both organic and inorganic pollutants. This mechanism of interaction with organic pollutants of DBC includes π-π EDA interaction, hydrogen bonding, and electrostatic attraction. However, the interaction mechanisms with inorganic pollutants involve ion exchange, surface complexation, and electrostatic attraction.

Because of DBC extended residence time in the marine environment and its role as a long-term aquatic carbon pool, as well as its distinctive chemical structure that facilitates interactions with heavy metal(loid)s and complex organic contaminants, DBC exhibits robust adsorption capabilities. This results in the transfer of electrons, which increases the breakdown and removal of contaminants such as imidacloprid, Hg(II), As(V), and 17β-estradiol (Fu et al., 2016; Coppola and Druffel, 2016; Fu et al., 2018). DBC plays numerous roles in the cycling of organic carbon (OC) in hydrological and geochemical systems, ultimately affecting the transportation and fate of nutrients and contaminants within soil and the marine environment (Coppola et al., 2022). DBC demonstrates its role as a vector and an efficient adsorbent for hydrophobic compounds within the ecosystem and has been shown to enhance the feasibility of BC consumption for the remediation of hydrophobic compounds from contaminated sediments and soils (Wu et al., 2022). The mobility of OCs in agricultural soils is likely influenced by the development and processes of DOC. DOC contains several functional groups, including the OH group, which can form H bonds with contaminants (Smith et al., 2013). Many non-ionic polar herbicides, including dicamba, 2,4,5-trichlorophenoxyacetic acid, and 2,4-dichlorophenoxyacetic acid, are primarily sorbed through H bonding. The characteristics of H bond formation and OCs among the COOR and carboxylic groups of the DBC regulate the movement of OCs in the soil (Dittmar et al., 2012). Figure 6 shows how DBC and pesticides interact through several processes, such as hydrophobic effects, which can reduce the movement of pesticides in the soil (García-Jaramillo et al., 2020). DBC may be considered a distinct type of DOC due to its complex aromatic structures and extreme environmental resistance. The various aromatic fractions that exhibit relatively high voltaic polarization are typically responsible for the strong correspondence of DOC with polar hydrophobic compounds (HOCs). This supports the interactions of Van der Waals forces with hydrophobic molecules, primarily controlling the risks and behaviors of HOCs over an extended period. The great affinity of DBCs for HOCs is facilitated by its molecular arrangements, allowing DBC to adopt a pseudo-micellar configuration through inter- and intramolecular interactions in water (Fu et al., 2018).

The domains of non-polar hydrophobic hydrocarbons accumulate in the core spheres of micelle-forming hydrophobics, which are accessible for HOC segmentation. The utilization of π-π and Van der Waals interactions by aromatic fractions facilitates self-adjustment. Although the hydrophilic spheres (such as functional groups or fractions that contain O and N) are primarily located at the DBC water interfaces (Yang et al., 2021), carboxylic groups may modify them through H bonding (Ambaye et al., 2020). Aromatic carboxylic acid groups have simple structures like DBC molecules and can readily form macromolecular assemblies due to the dipole–dipole interactions of the carboxylic acid groups (Lackinger and Heckl, 2009). For binary causes, DBC's high sorption capacity cannot be solely attributed to its higher aromaticity. Compared to other DOC with comparable aromatic C compounds, such as the 56% aromatic C in Aldrich HA, DBC exhibits a significantly higher PAH sorption affinity (Wang and Zhang, 2014), while Elliott soil HA compounds contain 46% aromatic C (Tanaka et al., 2005).

The interaction ability of PAHs with DBC is slightly higher compared to chlorinated benzenes with equal hydrophobicity but significantly lower p-electron density, as chlorinated benzenes are considered non-p-electron donors, suggesting a minimal contribution of π-π connection/assembling interactions. The formation of DBC featuring aromatic ring arrangements may dominate at high-temperature BC, whereas DBCs with chains of fatty carbons are likely more prominent at low pyrolysis temperatures of BC.

The investigation observed that DBC had a substantial impact on the binding curves of PHN with DBC, indicating that the chemical structure of DBC is important for PHN sorption (Fu et al., 2018). DBC, which has high aromaticity and lower molecular weights, is one of the more effective catalysts for the photodegradation and photo-transformation of anionic organic pollutants. DBC facilitated the photodegradation of diethyl phthalate via the generation of hydroxyl radicals (OH), metribuzin, and singlet oxygen (^1^O_2_) (Fang G. et al., 2017; Serelis et al., 2021). Additionally, DBC may enhance the photo-transformation of chlortetracycline and 17β-estradiol (Zhou et al., 2018; Tian et al., 2019). Furthermore, it has been demonstrated that DBC might enhance the photodegradation of 24 pharmaceutically active substances that are susceptible to one-electron oxidation by DBC (Wang et al., 2020). The interaction between DOC and trace metals varies depending on the size and molecular weight of DOC (Waqas et al., 2014). DOC's contact with metals decreases the toxicity and bioavailability of metal ions in the marine ecosystem (Hameed et al., 2023). DBC generated from BC bound to metallic element ions can be significantly affected by the temperature of pyrolysis, the chemical composition and level of DBC, as well as the chemistry of water, such as pH and the type and concentration of salt (Hameed et al., 2019). Previous research has shown that ionic strength and pH might impact the copper (Cu) and mercury (Hg) complexation mechanisms with DBC (Cao et al., 2004; Al-Reasi et al., 2011). The fundamental processes of metal attachment to DBC involve hydrogen bonding, electrostatic attraction, ion-exchange, and complexation. Heavy metals can transform into hydrolytic species and associate with inorganic ligands, including sulfates (${\text{SO}}_{4}^{2-}$) and chlorides (Cl), in the soil solution. The sizes of ligands and metals affect the binding of inorganic complexes and hydrolytic species (Lu and Allen, 2002). Hydrogen ions and cations exhibit equivalent binding affinities to complexes containing the same functional groups, which may interact with additional metals based on their levels and affinities for those metals (Chen et al., 2013). The complexation capability of DBC has been attributed to its functional groups and structural characteristics, such as amino, alcohol, and N-containing functional groups, as well as thiol, carboxyl, O–, S–, and phenolic groups, all of which are responsible for binding with metals (Pandey et al., 2003; Wu et al., 2007). Metal complexation with humic substances generally occurs at the phenolic and carboxylic sites (Hameed et al., 2019). Phenolic groups are usually associated with high-affinity sites; however, carboxylic groups are associated with low-affinity sites. Moreover, N-containing functional groups are considered less effective, even though they play a key role in metal binding (Inyang et al., 2016). Additionally, the molecular structure and quality of DBC may affect the cation binding mechanism. The protein-like ligand demonstrates a higher complexation affinity for Cu^2+^ than substances like HA. Previous studies have also observed that the binding of DOC with Cu^2+^ and Hg^+^ differs based on the structure of HA-like substances (Yamashita and Jaffé, 2008).

Huang et al. (2019) studied the characteristic interface between heavy metals and DBC associated with the interface affinities of Cd^2+^ and Cu^2+^ across various DBC fractions derived from the BC of rice straw and observed that the metal binding order is influenced by both DBC and metal fractions in a similar manner. Functional groups play an important role in the interface of heavy metals through ion-exchange mechanisms, surface complexation, and electrostatic attraction. For instance, ionic metals such as Pb^2+^ can transfer cations including Mg^2+^, Na^+^, Ca^2+^, and K^+^ (Lu et al., 2012) by interacting with the OH group to develop metal hydroxide species such as soluble or insoluble precipitates M(OH)_2_ and M(OH)^+^ of the DBC complex (Liu et al., 2019).

## 7 Future perspectives

Black carbon particles (BCPs) are residual organic compounds produced as a result of the combustion of fossil fuels and other biowastes. BCP has the potential to absorb light and contribute to atmospheric warming by generating a substantial amount of heat (Kruger et al., 2023). Recent studies have shown that DBC is an important portion of BC that is carried into the ocean due to its movement and conveyance by rivers (Coppola et al., 2018). The water heating behavior is adversely affected by the vigorous light-absorptive characteristics of DBC groups. The organic substances in aquatic environments absorb a wide range of wavelengths of light between the infrared and UV spectrum, with aromatic compounds exhibiting absorption at ~254 nm. The spectral and compositional analysis of DBC has revealed fewer aromatic properties than its parental biomass-based BC (Qu et al., 2016). However, DBC possesses greater aromatic characteristics than other organic compounds in water bodies, suggesting its significant role in heat generation due to high absorption capacities (Wagner et al., 2018). Research is needed to address concerns about how the light-absorbing and heat-generating properties of DBC in water might impact the growth of microalgae. The future research directions regarding the influence of DBC on microalgal growth and development are suggested as follows:

- **Quality assessment:** The inhibitory or stimulatory concentration ranges of DBC should be determined through quantitative analysis for the growth of microalgae. The range of DBC amounts affecting various species of microalgae should be studied to comprehend the dose–response associations.
- **Species-specific responses:** The species-specific responses of microalgae to DBC need to be explored. Each species may possess different strategies for adapting to DBC.
- **Mechanistic understanding:** The underlying mechanisms and processes involved in the association between microalgae and DBC should be investigated. This can be achieved by analyzing the physiological, biochemical, and molecular behavior of microalgae during their exposure to DBC.
- **Environmental context:** The different environmental factors, including nutritional composition, temperature, and light intensities and wavelengths, can be studied to determine their influence on the effect of DBC on microalgal growth. The behavior of microalgae could also be affected by varying these factors.
- **Long-term effects:** The continuous long-term impact of DBC on growth, behavior, and community variations can be evaluated to better understand their associations. This would help us explore the strategies for adaptation and acclimatization of microalgal species to DBC.
- **Ecosystem-level studies:** Broader-scale ecosystem research can be conducted to evaluate and understand the complex interactions among DBC, microalgae, and other organisms in the environment.
- **Integration with climate change research:** The impact of climate change can be explored on a broader scale to understand the associations between microalgae and DBC under varying climatic conditions. The carbon cycle and greenhouse gas emissions may be affected by these factors.
- **Development of mitigation strategies:** The adverse effects of DBC on the growth of microalgae can be reduced by applying various approaches, such as enhancing microalgal resistance or implementing techniques that mitigate the negative impacts of DBC in aquatic ecosystems.

## 8 Conclusions

Although often regarded as environmental pollutants, some carbonaceous substances can be carefully managed to support sustainable development goals. DBC, a small fraction of BC, is gradually released into various environmental sources, including surface water, soil, and the marine environment, through infiltration and runoff. The differences in the release kinetics of DBC and nutrients from BC across terrestrial and aquatic environments, as well as at the soil–water interface, need to be studied. DBC in the aquatic environment absorbs sunlight, increasing water temperature and interacting with other pollutants, which may mitigate its toxicity. Despite limited literature, this review suggests that the introduction of BC, particularly DBC, can enhance microalgal growth. Furthermore, the cultivated microalgae might play important roles in carbon cycling and sequestration. The precise role of DBC in microalgal growth is unclear, warranting detailed investigation and potential mitigation strategies to counteract any negative effects of BC and DBC on microalgal growth.
